# How to individualize therapy after failing milestones in chronic myeloid leukaemia: weighting late response and early death from CML against risk of alternative therapies

**DOI:** 10.1038/s41375-024-02139-4

**Published:** 2024-01-13

**Authors:** Rüdiger Hehlmann, Michael Lauseker

**Affiliations:** 1 Medizinische Fakultät Mannheim, Universität Heidelberg, Mannheim, Germany; 2 ELN Foundation, Weinheim, Germany; 3 Institut für Medizinische Informationsverarbeitung, Biometrie und Epidemiologie – IBE, Medizinische Fakultät, LMU München, München, Germany

**Keywords:** Chronic myeloid leukaemia, Randomized controlled trials

## To the Editor:

We thank Prof. Kantarjian for his Commentary (What is the impact of failing to achieve TKI therapy milestones in chronic myeloid leukemia. *Leukemia*. 2023 37:2324-5) to our recent article [1]. We would like to add data on late responses and early deaths from CML to help with therapy decisions [2].

Table 1 quantifies late responses and early deaths from CML in failing subjects from CML-study IV [3]. Although the samples are all the same subjects the Table shows numbers of non-responders at the 10% *BCR::ABL1*^IS^ level decrease steadily from 28% at 3 months to 12% at 6 months, 8% at 1 year and 5% at 2 years. When following the actual subjects in the 3 months sample our conclusion is similar: There are always late responders. However, failures at the later landmarks are associated with a lower probability of a late response. Amongst responders there are no data time of response correlates with prognosis.

**Table 1:** Non-responders and early deaths after 3, 6, 12 and 24 months in 1342 CML patients under imatinib based treatment.

Months	3	6	12	24
Patients at risk	805	891	861	755
>10% BCR::ABL1^IS^	223 (28%)	104 (12%)	65 (8%)	37 (5%)
Early deaths within 12 months after landmark: after progression / total	2/4	7/7	8/9	3/4

Death by CML is defined as death after progression.

Numbers of deaths within 1 year after the landmarks are low. Only 2 subjects failing the 3 months landmark with *BCR::ABL1*^IS^ > 10% died of CML within the following year. 7 subjects failing at 6 months died of CML, 8 failing at 1 year and 3 failing at 2 years.

Table 2 shows the rate of early non-responders is higher in young subjects, but early deaths are not age-related.

**Table 2:** Non-responders and early deaths after 3, 6, 12 and 24 months by age more or less than 60 years.

Age	<60 years				≥60 years			
Months	3	6	12	24	3	6	12	24
Patients at risk	537	597	567	490	268	294	292	265
>10% BCR::ABL1^IS^	164 (31%)	67 (11%)	38 (7%)	25 (5%)	59 (22%)	37 (13%)	27 (9%)	12 (5%)
Early deaths within 12 months after landmark	3	4	4	3	1	3	5	1

It follows that subjects failing the 10% *BCR::ABL1*^IS^ milestone at 3 and 6 months have high probabilities of late responses and a low risk of early death from CML.

A **1-year failure landmark** is supported by data indicating that in subjects not reaching the 10% *BCR::ABL1*^IS^ milestone at 3 months cumulative incidence of MR^3^ at 5 years is 83% whereas the competing event of death at 5 years has a cumulative incidence of only 9%. Similarly, in subjects failing the 6 month *BCR::ABL1*^IS^ milestone cumulative incidence of an MR^3^ at 5 years is 74%, whereas cumulative incidence of death is 10%. This is different at 1 year whereby in subjects not reaching 10% *BCR::ABL1*^*I*S^ 5-year cumulative incidence of an MR^3^ decreases to 27% and cumulative incidence of death is 30%.

Our analyses are of subjects receiving imatinib. Whether our conclusions apply to subjects receiving other tyrosine kinase-inhibitors needs study.

In conclusion, people failing 10% *BCR::ABL1*^IS^ at 3 or 6 months need critical evaluation of benefits and risks of alternative interventions. This is because the likelihood of a response during the following few months is high and risk of death from CML low. Individualized therapy decisions are desirable. The challenge is to accurately identify the few people early who will never respond and progress.
